# Disrupting Effects of Osteogenesis Imperfecta Mutations Could Be Predicted by Local Hydrogen Bonding Energy

**DOI:** 10.3390/biom12081104

**Published:** 2022-08-11

**Authors:** Shumin Qiang, Cheng Lu, Fei Xu

**Affiliations:** Ministry of Education Key Laboratory of Industrial Biotechnology, School of Biotechnology, Jiangnan University, Wuxi 214122, China

**Keywords:** collagen, heterotrimer, hydrogen bond, MD simulation, OI mutation

## Abstract

Osteogenesis imperfecta(OI) is a disease caused by substitution in glycine residues with different amino acids in type I collagen (Gly-Xaa-Yaa)n. Collagen model peptides can capture the thermal stability loss of the helix after Gly mutations, most of which are homotrimers. However, a majority of natural collagen exists in heterotrimers. To investigate the effects of chain specific mutations in the natural state of collagen more accurately, here we introduce various lengths of side-chain amino acids into ABC-type heterotrimers. The disruptive effects of the mutations were characterized both experimentally and computationally. We found the stability decrease in the mutants was mainly caused by the disruption of backbone hydrogen bonds. Meanwhile, we found a threshold value of local hydrogen bonding energy that could predict triple helix folding or unfolding. Val caused the unfolding of triple helices, whereas Ser with a similar side-chain length did not. Structural details suggested that the side-chain hydroxyl group in Ser forms hydrogen bonds with the backbone, thereby compensating for the mutants’ decreased stability. Our study contributes to a better understanding of how OI mutations destabilize collagen triple helices and the molecular mechanisms underlying OI.

## 1. Introduction

Collagen is the most abundant protein in humans and accounts for 30% of all protein. It provides tensile strength for skin, bones and blood vessels [1,2,3,4]. The sequence of collagen contains unique repeats of Gly-X-Y, where the X position is usually occupied by proline (Pro) and the Y position is usually (2S,4R)-4-hydroxyproline (Hyp) [5,6]. Three collagen chains are intertwined to form a characteristic triple helix secondary structure. The structure is defined as a homotrimer where all three chains are identical; otherwise, it is called a heterotrimer. Each polypeptide chain is offset by one amino acid, which can present Gly in every cross-section of the triple helices [7]. Under this arrangement, the amide protons of Gly form hydrogen bonds (H-bond) with the carbonyl oxygen of the X residue of the adjacent peptide chain. The formation of a series of ladder-like H-bonds from the N-terminus to the C-terminus of collagen is the main driving force in stabilizing the triple helix structure [3,8]. Despite these facts, collagen’s triple helix structural change has not been studied in relation to H-bond destruction.

There are 28 types of collagen in humans, the largest of which is type I collagen, with a heterotrimeric structure consisting of two α1 chains and one α2 chain [4]. Medical research has found that when a single point mutation occurs in type I collagen, Gly is often replaced by other residues, which leads to a variety of connective tissue diseases, including osteogenesis imperfecta (OI) [2,9,10,11]. Symptoms in patients with OI vary widely, ranging from mild multiple fractures to severe perinatal lethality [12,13]. A single-base substitution in a Gly codon can lead to any of the eight different residues: Ser, Cys, Ala, Val, Asp, Glu, Arg and Trp [14,15,16]. Current studies have shown that the identity of the residue replacing Gly appears to be closely related to the clinical severity of OI cases; however, the links between mutation types and disease symptoms are unclear.

In previous studies, OI mutation sites were inserted into a highly stable host model to construct mutants. The effects of mutations on collagen triple helix structures were characterized by X-ray diffraction, nuclear magnetic resonance (NMR) spectroscopy and circular dichroism (CD) experiments [15,17,18,19,20,21,22,23,24,25,26,27]. This host model is called a collagen model peptide, often containing Gly–Pro–Hyp repeating sequences that can form stable helix structures in solution. Different types of OI mutation motif sequences have been introduced into the homotrimer to disrupt helix structures, resulting in a significant reduction in thermal stability. It was found that mutations located on different chains can trigger different disruptive effects [15,28,29]. A typical Gly → Ala mutant (POG)4POA(POG)5 (PDB ID 1CAG) was investigated by NMR. The results showed that Ala substitution led to a decrease in melting temperature (*T*_m_) from 60 to 29 °C [30]. NMR studies have demonstrated differences among the three chains’ substitution. High NH temperature gradients in the tailing chain indicated that Ala caused H-bond destruction, whereas the other two Ala still had low NH temperature gradients [31]. In order to better mimic the composition of type I collagen, in recent years, with the development of rational heterotrimer synthesis technology, scientists began to study OI mutations with heterotrimers as the host models [32,33,34,35]. When Gly → Ala was introduced in the AAB model, 5 H-bonds were destroyed, caused by the mutation in chain B [36]. In contrast, while the mutation occurred in chain A of the ABB model, only 1 H-bond was destroyed [37]. While there are studies on heterotrimers, little research has been conducted to reveal chain specific mutation effects.

Molecular dynamics (MD) simulations have now become a new way to characterize the effect of OI mutations on collagen’s triple helix structure in the host–guest model. In a study, changes in backbone H-bonds conformation showed one series of the H-bonds near the mutation site were disrupted, and the other part was remodeled into water-mediated H-bonds. At the same time, different from the rod-like triple helical in the crystal, the molecular tortuosity was significantly increased [29,38,39,40]. MD simulations in a 1014-residue type I collagen model containing several OI mutations showed some of the structures near the mutation site appeared to be complete triple helixes, whereas some were partially unfolded, indicating differences in the structural damage were caused by the mutations in different regions [41]. Although these studies suggest that Gly mutations in the collagen sequence affect the triple helix structure, the disruptive effects of various amino acids on the local area require further research.

Here we introduce serine, valine, aspartic acid and arginine to replace Gly in the middle of three chains in the *abc* heterotrimer, respectively. The disruptive effects of the mutations were characterized by biophysical experiments and molecular dynamic simulations. The relationship between the reduced stability and conformational change was studied, which will advance our understanding of collagen folding and related diseases.

## 2. Materials and Methods

Peptide design: Our laboratory has successfully constructed a heterotrimer model with three different chains, named *abc*. This collagen-model peptide, as a platform, allows us to study changes in single-chain mutants with the introduction of OI mutations. Based on previous experiments with Ala mutants, the 17th amino acid of each chain was chosen as the mutation site to reduce the effect of salt bridge change on thermal stability. In this way, the influence of the H-bond swing due to the N- and C-termini was eliminated.

The peptides were synthesized using solid-phase FMOC chemistry and purified to 95% purity by reverse-phase HPLC with mass spectrometry at GL Biochem Ltd. (Shanghai, China). The N- and C-termini were uncapped. The sequences of the characterized peptides are listed in Table 1; the substituted Gly is highlighted in red.

Circular Dichroism (CD) Spectroscopy: The peptides, as listed above, were dialyzed in deionized water, freeze dried, weighed and then redissolved in 10 mM phosphate buffer at pH 7.0 to make stock solutions. The stock solutions of A, B, C and the corresponding peptides with the designed mutations were further diluted to 0.2 mM, mixed up at 1:1:1 ratio, heated at 80 °C for 10 min and then incubated at 4 °C for ~24 h before CD. The CD experiments were performed in a Chirascan instrument (Applied Photophysics Ltd., London UK) using optically matched quartz cuvettes with a path length of 0.1 cm (Model 110-OS, Hellma, Müllheim, Germany). Three independent wavelength scans were used from 190 to 260 nm at 4 °C with a 0.5 nm increment per step and a 0.5 s averaging time. For temperature melting experiments, the data were recorded every 1 °C/step with a 6-min equilibration time from 4 to 80 °C. The temperature melting curves were acquired by monitoring the ellipticity at 225 nm. The apparent melting temperature, Tm, was estimated by the equation below:

(1) $$F\left(T\right)=\frac{\theta \left(T\right)-\theta_{U}\left(T\right)}{\theta_{F}\left(T\right)-\theta_{U}\left(T\right)}\;$$

where θ(T) is the observed ellipticity. (***T***) and (***T***) are estimated ellipticities derived from linear fits to the folded and unfolded baselines. The melting temperature is estimated as ***T***, where ***F***(***T***) = 0.5.

Molecular dynamics: The coordinates of chains *a*, *b* and *c* were used as the initial structure for molecular dynamic simulations. The structure was placed in a truncated dodecahedron periodic box of the explicit TIP3P water model with approximately 39,291 water molecules. The distance from the surface of the box to the closest atom of the solute was set to 10 Å. The simulation was carried out in the Amber99sb*-ILDN force field with GROMACS. The lengths of bonds involving hydrogens were constrained, allowing for a 2 fs time step. Long-range electrostatic interactions were evaluated in reciprocal space using the particle-mesh Ewald method [42] with a maximum spacing for the FFT grid of 1.2 Å and an interpolation via a sixth-order polynomial. The minimal cut-off distance for electrostatic and van der Waals interactions was set to 12 Å. The system was relaxed to a local energy minimum using the steepest descent method [43]. Subsequently, a 10 ns NPT and a 100 ns NVT simulation were conducted. A temperature of 298 K was maintained via the velocity rescaling algorithm (0.1 ps relaxation time), and the pressure P = 1 bar was controlled using the weak coupling method of Berendsen.

Cα triangles: The movement of collagen is regarded as the displacement of a series of triangles, and the vertex of the triangle is the Cα of Gly and the adjacent chain (A1, A2). The calculation process is as follows:

Gly (*x_1_*, *y_1_*, *z_1_*), A1 (*x_2_*, *y_2_*, *z_2_*), A2 (*x_3_*, *y_3_*, *z_3_*). Here, A2 pointed towards A1 determines the *y*-axis direction.$\underset{a}{\to }\;$= (*x_a_*, *y_a_*, *z_a_*), where

$$x_{a}=x_{2}-x_{3}\;y_{a}=y_{2}-y_{3}\;z_{a}=z_{2}-z_{3}$$

Origin coordinates O (*x_o_*, *y_o_*, *z_o_*):

$$x_{o}=\frac{x_{2}+x_{3}}{2},\;y_{o}=\frac{y_{2}+y_{3}}{2},\;\;z_{o}=\frac{z_{2}+z_{3}}{2}.$$

Additionally, a vector $\underset{b\;}{\to }\;$on the plane, $\underset{b\;}{\to }$ = (*x_b_*, *y_b_*, *z_b_*):

$$x_{b}=x_{1}-x_{o},\;y_{b}=y_{1}-y_{o},\;z_{b}=z_{1}-z_{o}$$

The direction of the *z*-axis $\underset{c\;}{\to }$ = (*x_c_*, *y_c_*, *z_c_*)

$$x_{c}=y_{b}z_{a}-z_{b}y_{a},\;y_{c}=x_{b}z_{a}-z_{b}x_{a},\;z_{c}=x_{b}y_{a}-y_{b}x_{a};$$

The direction of the *x*-axis $\underset{m\;}{\to }$ = (*x_m_*, *y_m_*, *z_m_*)

$$x_{m}=y_{a}z_{c}-z_{a}y_{c},\;y_{m}=x_{a}z_{c}-z_{a}x_{c},\;z_{m}=x_{a}y_{c}-y_{a}x_{c};$$

The coordinates of any point *i*(*x_k_*, *y_k_*, *z_k_*) projected on this plane are *i* (*x_i_*, *y_i_*, *z_i_*)

$$x_{i}=x_{k}-x_{o},\;y_{i}=y_{k}-y_{o}.\;$$

H-bond energy: The amide protons of Gly (N-H) in the collagen triple helix can form a H-bond with the carbonyl oxygen (C=O) of the amino acid at the X position of the adjacent chain. In a simulation, an H-bond is defined as the interatomic distance *R* between the donor and acceptor less than 3.5 Å, and simultaneously, the hydrogen–donor–acceptor angle α of less than 30° [44]. The H-bond energy was calculated as below:

(2) $$E_{\mathrm{HB}\;}{=D}_{0}\left[5{\left(\frac{R_{0}}{R}\right)}^{12}-6{\left(\frac{R_{0}}{R}\right)}^{10}\right]F(\theta )$$

where $R_{0}$ is the mean distance in a well-folded H-bond of *abc*. Here $R_{0}$ = 2.9 Å, *D*_0_ is the well depth and *R* is the interatomic distance between the nitrogen and oxygen. The restrictive angle-dependence term, $F\left(\theta \right){=\cos}^{2}{(\theta )\cos}^{2}\left(\varphi \right)$, is *sp*^3^ *donor--sp*^2^ *acceptor* hybridization-dependent, where the angle θ is the N_Gly_-H_Gly_-O_Xaa_ angle and the angle φ refers to the C_Xaa_-O_Xaa_-H_Gly_ angle.

Statistical analysis: The experimental data from all studies are presented as means ± standard deviation (SD). One-way analysis of variance (ANOVA) was used to test the experimental results. *p* < 0.05 was considered as statistically significant.

## 3. Results

### 3.1. Thermal Stability of Collagen with Gly Mutations

**Figure 1:**
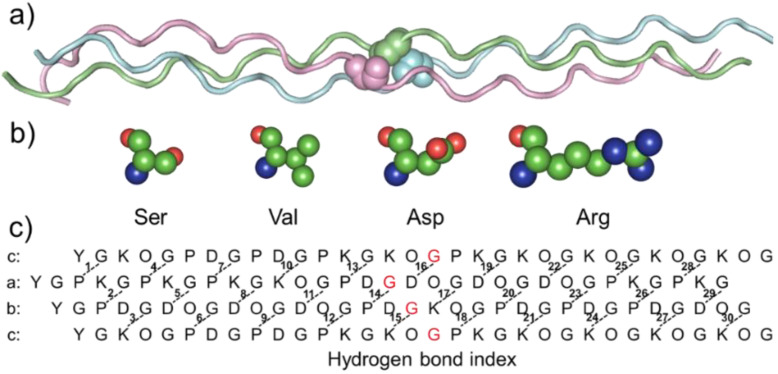
(**a**): Cartoon-loop diagram of *abc* (PDB ID:5YAN) peptide models is represented with the N-terminal at the left. The mutation residue is shown in spheres; (**b**) sphere model of mutant amino acid; (**c**) sequence of *abc* and the H-bond pattern. The mutation residue is marked in red.

To investigate the impact of mutations on collagen structure, a previously designed register and composition controlled *abc* heterotrimer was used as a host model (Figure 1a). To explore the difference in reducing thermal stability caused by distinct side-chain amino acids, four amino acids were introduced into the mutation sites (Figure 1b,c). To simplify it, *abc* heterotrimeric helices with mutant were named after the amino acid and chain—for example, a17S, b17S and c17S.

**Figure 2:**
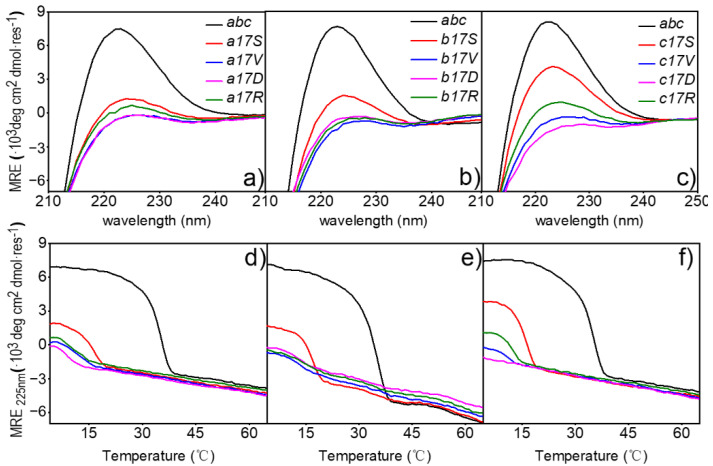
Thermal stability of *abc* and 4 mutants measured by CD spectroscopy. (**a**–**c**) Far-UV CD spectra (**d**–**f**). Thermal denaturation of host-guest peptide. Mutants are indicated by solid lines on the top right; peptides are indicated with different colors.

To better understand the implications of mutations on structure, the triple-helical conformation and stability of mutant collagen were examined. Far-UV CD spectra recorded at 4 °C in PB showed a maximum of varying magnitudes at 225 nm for all host-guest peptides examined (Figure 2a–c). The host peptide *abc* showed a value of [*θ*]_225_ ≈ 7113 ± 276 deg∙cm^2^∙dmol^−1^ in standard conditions, indicating a stable triple-helical conformation. The corresponding values of peptides Ser mutant were lower but still showed a trend in self-assembly, with values of [*θ*]_225_ ≈ 1284, 1696, 3861 deg∙cm^2^∙dmol^−1^ in chain *a*, *b* and *c*, respectively. The absorption values of the other three mutants at this wavelength were all near 0 deg∙cm^2^∙dmol^−1^, indicating that no helix was formed.

To clarify the effects of the mutation sites on triple-helical stability, thermal transition curves were determined by monitoring the change in ellipticity at 225 nm on the heater (Figure 2d–f). The host peptide *abc* and peptides of three Ser mutants showed a sharp thermal transition from the trimer (native) to monomer (denatured) state, with the *T*_m_ values of 34.47 ± 0.3 °C [35] and 15.78 °C in a17S, 16.67 °C in b17S and 15.89 °C in c17S, respectively (Table 2). In contrast to Ser mutants, a non-obvious change in the curve of the ellipticities with the increase in temperature was recorded for the Arg, Val and Asp mutations, suggesting a denatured conformation, even at low temperatures. In contrast to Ser mutants, the ellipticities curve changes for Arg, Val and Asp mutations were not evident with increasing temperature, suggesting a denatured conformation, even at low temperatures (Figure 2d–f). When the full-wavelength scanning absorption value was below 0 or the fitting temperature was below 10 °C, it was considered that no triple helix was formed. Therefore, the triple-helix did not form in the Arg, Val and Asp mutants.

### 3.2. H-Bond Energy Destruction Due to Mutation

**Figure 3:**
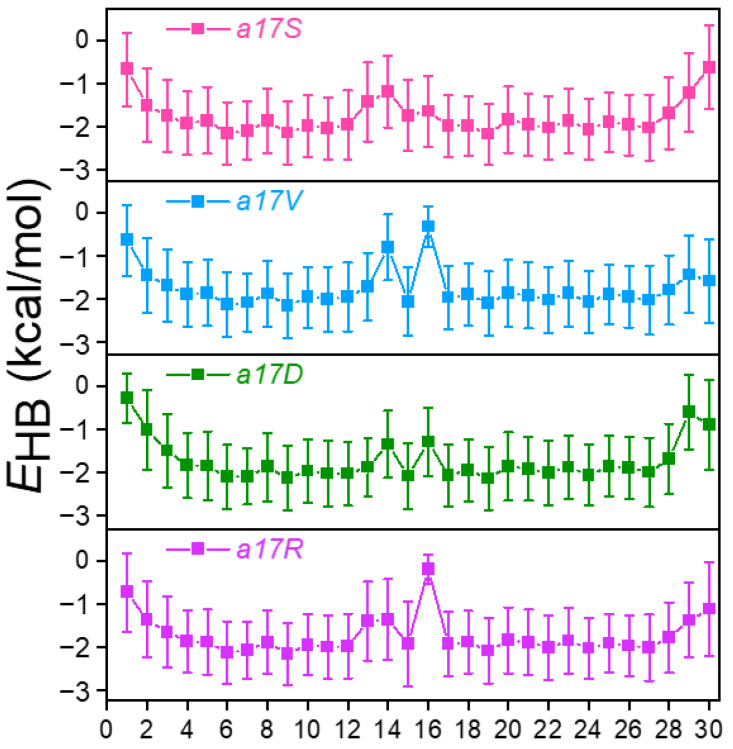
*E_HB_* of H-bond binding after Gly of chain *a* 17th site was mutated. Indexes 1–30 represent the position of each H-bond in the collagen.

Average inter-chain H-bond energy (*E_HB_*) in various mutants, and *abc* was extracted from the MD trajectories and calculated (Figure 3 and Figure S1). In *abc*, since the N and C terminals were more flexible when exposed to the solvent, the energies of the H-bonds 1st–4th and 29th and 30th were lower (Figure S1a). In the region where the collagen structure was stable, the energy of H-bonds was stabilized at 2.0 kal/mol, indicating that H-bonds were well-formed. Correspondingly, in previous experimental studies, it was found that the H-bond energy in the collagen helix was 2.0 kcal/mol [3,45] Meanwhile, the probabilities ($P_{\mathrm{HB}}$) of H-bonds were calculated (Figure S2). Then the $E_{\mathrm{HB}}$ in the well-folded region was compared with the $P_{\mathrm{HB}}$, defined as$\;{\;E}_{\mathrm{HB}}^{\mathrm{per}}{=E}_{\mathrm{HB}}{/P}_{\mathrm{HB}}$. The results showed that the value of${\;E}_{\mathrm{HB}}^{\mathrm{per}}$ was close to two, suggesting that a stable H-bond in *abc* contains about 2 kcal/mol. In order to avoid interference from N- and C-termini, 6th–25th H-bonds were chosen for analysis.

Around the substitution site, with different amino acids introduced, there was obvious destruction of the H-bond in all mutants (Figure 3 and Figure S1b,c). When substitution occurred in chain *a*, more than one H-bond was disrupted (Figure 3). In a17S, the 14th H-bond demonstrated the greatest disruption with an average *E_HB_* increase from −2.0 to −1.1 kcal/mol, and the 13th *E_HB_* decreased by 0.6 kcal/mol. In a17V and a17D, the 14th and 16th H-bonds were broken; the *E_HB_* increased from 0.8 to 1.2 kcal/mol. Unlike these two mutants, we observed that 3 H-bonds were disrupted in a17R (13th, 14th and 16th). Substitution in the chain also broke one salt bridge in the side-chain (Figure S3), leading to more damage to the local structure.

Differently from the substitution in chain *a* that caused destruction of multiple H-bonds, substitution in chain *b* and *c* only destroyed one H-bond significantly (Figure S1b,c). In b17X, the 17th H-bond was disrupted with the *E_HB_* in range −0.1 to −0.40 kcal/mol. Similar to the chain *b* mutant, the substitution in chain *c* affected only one H-bond as well (18th). These results indicate that the destruction of the helical structure is not only determined by amino acid type, but is also related to the sequence environment around the substituted site.

### 3.3. Cα-Triangle Reference Area

The repetition of the G–X–Y sequence in collagen results in its trajectory appearing like the displacement of a series of triangles, with each side length representing the folding degree of the region. To describe the local regional structure changes after substitution, and to avoid the oscillating effects of the N-terminal and C-terminal, the trajectory was extracted at 5000 frames, and the mean side-length of the 14–18th triangle was calculated.

**Figure 4:**
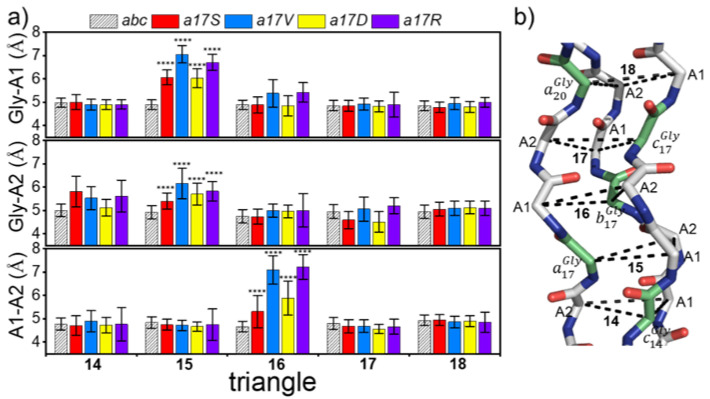
The side length of the Cα triangle after substitution. (**a**) The side lengths of the 14th–18th triangle of *abc* and chain a mutants. *abc* is represented by a bar graph with right-slanted lines, and other mutants are shown in various colors, as indicated in the above. (**b**) 14th–18th triangle structure of *abc*; Gly is shown in lime green; A1 and A2 sites are marked in the graph. Statistical significance was calculated using one-wany ANOVA. Values are mean ± SD. **** *p* < 0.0001, versus *abc* alone.

Figure 4a shows the side-lengths of five triangles in *abc* and chain *a* mutants; the position of the corresponding triangle in triple helix is shown on the right; and Gly is marked in green. The side lengths of the triangles in *abc* were 4.81 ± 0.09 Å, suggesting that in the absence of mutations, the side lengths and the structure of the triangles were very stable. In all three chains, the mutations resulted in an increase in the A1–A2 length of the next triangle at the C-termini (Figure 4 and Figure S4). Meanwhile, the Gly mutations in chains a and b also led to an increase in the length of Gly-A1 prior to the N-termini. Among the four mutant amino acids, the side length of the triangle in both Val and Asp was damaged greatly. For example, in all chains of Val, the mutation resulted in the decrease in side length to 7 Å (Figure 4 and Figure S4). The Ser mutant demonstrated the least disruption compared to the other amino acids, with an increase of around 5.5–6.5 Å in the triangle length. In addition, the introduction of Asp showed different effects on the three chains: the maximum triangle length of a17D increased to 6Å; b17D and c17D increased to around 7 Å.

These results indicate that larger side-chains appear after the introduction of Gly-X mutation, leading to an increase in the distance between chains near the mutation site and the destruction in the local structure of collagen.

### 3.4. Side Chain Pattern in Collagen-like Peptide Helix

Although the increase in inter-chain distance may indicate disruption of the triple helix structure, how specific side-chains are distributed in collagen requires further study. Here we took the plane formed by the triangle as the projection plane, and then projected the carbon atoms in the amino acid side-chain to the plane to study the motion state of the side-chain. At the same time, the distributions of nitrogen atoms and hydrogen atoms in the most disrupted H-bonds in the mutants and the conventional H-bonds in *abc* were also projected on this plane. The changes of H-bonds before and after were compared.

**Figure 5:**
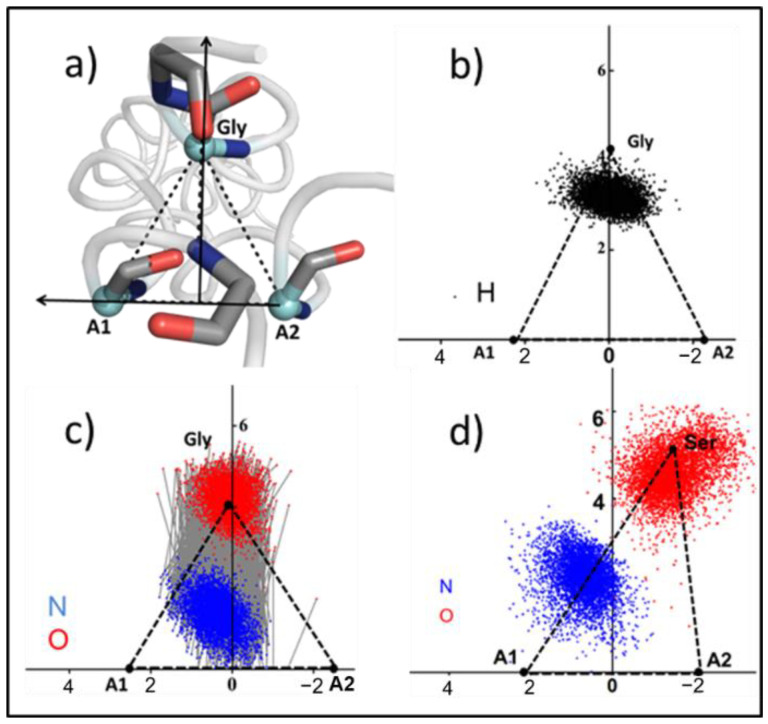
The distributions of nitrogen, oxygen and hydrogen atoms in H-bonds. (**a**) Top view of the Cα triangle. (**b**) The distribution of hydrogen atoms in a stable triangle. (**c**) The distribution of nitrogen and oxygen atoms in a stable H-bond. (**d**) The distribution of nitrogen and oxygen atoms in a broken H-bond in a17S. Hydrogen atoms are shown in black, nitrogen atoms are shown in blue, and oxygen atoms are shown in red.

Some of the scatter plot results are shown in Figure 5 and Figure S5. Gly, A1 and A2 are the vertices of the previous triangle (Figure 5a). In *abc*, the distribution of hydrogen atoms fell mostly inside the triangle, and the H-bond was also approximately perpendicular to the A1–A2 side (Figure 5b,c). However, due to the increase in the length of Gly-A1 in the a17S, the corresponding Gly position was shifted from its stable position, and the side length of A1–A2 was stable (Figure 5d). The distributions of H-bond atoms in b17S and c17S are shown in Figure S4. Therefore, the oxygen atom shifted correspondingly with the shift in the upper vertex, leading to an increase in H-bond distance, which is consistent with the increase in H-bond energy.

**Figure 6:**
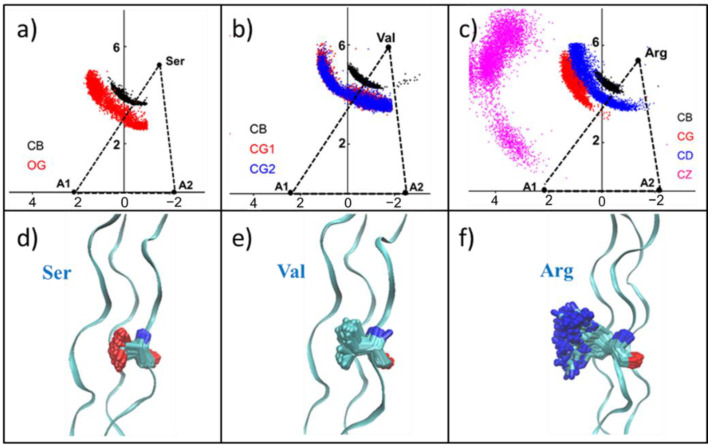
The distribution of mutant amino acid side-chains. (**a**–**c**) The distribution of heavy atoms in the side-chain in the triangular plane of Ser, Val and Arg. (**d**–**f**) Cartoon model of mutations with amino acids represented as sticks: oxygen (red), nitrogen (blue), carbon (cyan).

For the Ser mutation, the carbon atom (black) of the side-chain methyl group and the oxygen atom (red) of the hydroxyl group distribution are shown in Figure 6a and Figure S6a,d. Since -CH_2_-OH has a short side-chain length and a hydrophilic group -OH, some of its side-chains may be contained within the collagen helix region. Thus, the internal space of the triple helix was enlarged, and then the collagen structure was destroyed. Val mutants showed a similar distribution with Ser mutant, which was consistent with the similarity in the side-chain lengths of them (Figure 6b and Figure S6b,e). Moreover, because of the hydrophobicity of the Val side-chain, it has a tendency to be wrapped inside the collagen. The two methyl groups of the Val side-chain -CH-(CH_3_)_2_ demonstrated the same distribution, indicating that CG_1_ and CG_2_ have the same properties.

As the length of the side-chain increased, the distribution of heavy atoms in the side-chain of Asp and Arg showed another pattern, and all the side-chains were exposed in the solution (Figure 6c and Figure S6c,f). This result suggests that large side-chains cannot be located inside the triangle formed by Cα due to space obstruction.

### 3.5. Compensating H-Bond in Ser Mutation

Despite Ser and Val mutants exhibiting similar changes in H-bond and side-chain atomic distribution, these two mutants possessed huge differences in thermal stability characterization. To investigate this difference and the reason for the observed thermal stability of Ser, the possibility of side-chain H-bond under the presence of -OH in the Ser side-chain was taken into consideration.

**Figure 7:**
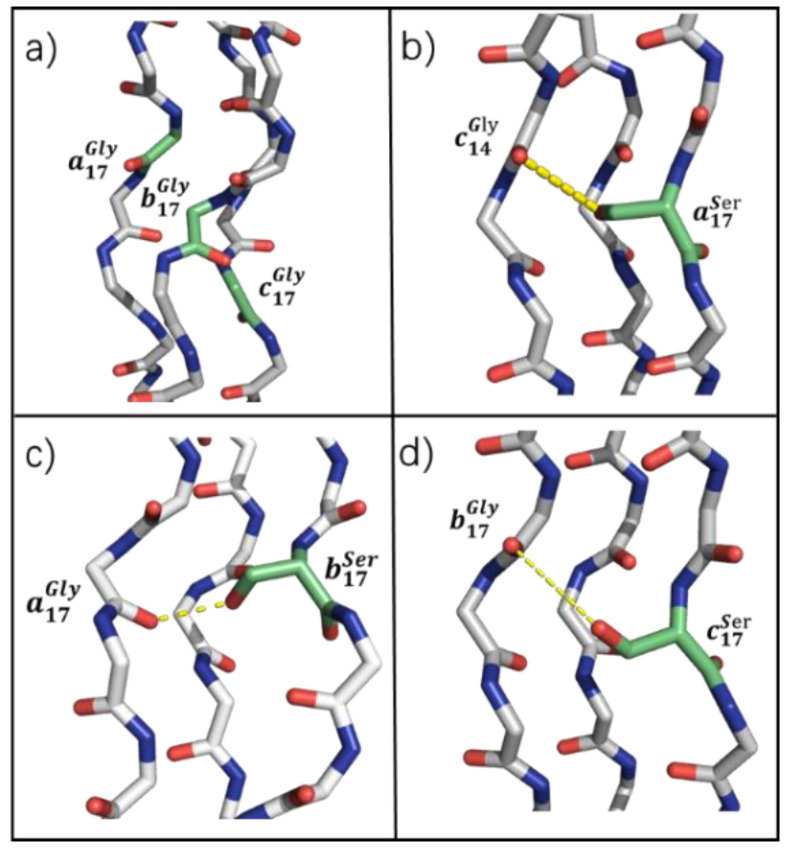
Snapshots of a section of the simulated collagen-like peptide triple-helix show the new direction of the H-bond compared to the original *abc*. The H-bond was formed between the Ser O-H and the Gly O on the adjacent chain, shown with a yellow dash. (**a**) No compensatory H-bonds in *abc*; (**b**) Compensatory H-bonds in a17S formed between 17th Gly in chain *a* and Ser in chain *a*; (**c**) Compensatory H-bonds in b17S formed between 14th Gly in chain *c* and Ser in chain *b*; (**d**) Compensatory H-bonds in c17S formed between 17th Gly in chain *b* and Ser in chain *c*.

It is shown that the -OH carried by Ser can form compensatory H-bonds with Gly in adjacent chains (Figure 7b–d). For example, Ser in chain *a* could act as a hydrogen donor, and the 14th Gly in chain *c* acts as a hydrogen accepter to form a compensatory H-bond (Figure 7b). $P_{\mathrm{HB}}$ of chain *a*’s compensatory H-bond is 0.40, and the $P_{\mathrm{HB}}$ of Ser in chains *b* and *c* with the 17th Gly in the adjacent chain are 0.38 and 0.49, respectively.

**Figure 8:**
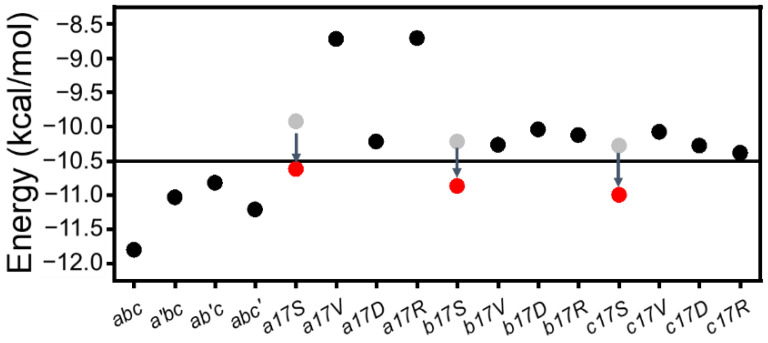
$E_{HB}$ of 2 G–X–Y regions. The $E_{HB}$ of the six main-chain H-bonds are represented by gray and black dots, and the local energy of the Ser mutant changes from gray to red after the compensation effect.

Due to the existence of G–X–Y repeating units in the collagen sequence, it is of interest how the G–X–Y unit in the mutant region could stabilize the triple helix structure. In order to investigate this question, the sum *E_HB_* of 6 H-bonds and Ala mutant (not published) was calculated (Figure 8). The results showed that after the single-point insertion of Ala, the energy increased from the initial −12 kcal/mol to around −10.5 kcal/mol. CD experiments also demonstrated that the substitution of Ala maintained a stable triple helix structure. When Gly was substituted with other amino acids, the sum of the energy increased to more than −10.5 kcal/mol, where the triple helix structure was disrupted. While adding compensatory H-bonds, the energy of this region dropped to −10.5 kcal/mol again (Figure 8); thus, Ser mutations still maintained the triple helix structure. The observed clinical severity for different residues indicate that Asp, Val and Arg may lead to more severe phenotypes than Ser [11], which is consistent with our research.

## 4. Discussion

The conformation and thermal stability of Gly substitution on the triple-helix were observed within 12 heterotrimers. Results showed that, although Gly substitutions could cause various conformational changes dependent on substituting residue identities, the stability of the triple helices should be mainly affected by the loss of backbone H-bonds. Various conformational changes occurred when Gly was substituted by side-chains of different sizes. Smaller side-chains, such as Val and Ser, could be inserted into triple helices or exposed to surfaces, and larger side-chains of Arg, were well exposed to solution. These conformational changes are also associated with the chemical environment surrounding the substitution site. In chain *a* substitution, an inter-chain salt bridge was severely disrupted by the large side-chain of *aa*. In Gly → Ser, the side-chain hydroxide group of Ser formed a new H-bond with the backbone amide group of Gly.

Here, a threshold value of local (mutations site 13th–18th H-bonds) H-bonding energy (=−10.5 kcal/mol) could be a good indicator of triple helix folding, which roughly corresponds to 0.75 H-bond loss in our system. The destruction of $E_{HB}$ can be caused by either severe disruption of a single H-bond or partial loss of multiple H-bonds. Notably, the newly formed side-chain backbone H-bond in Gly → Ser substitution could compensate for the loss of the backbone H-bonds and help maintain the stability. The compensating H-bond was also observed in AAB-type collagen heterotrimer containing Gly → Ser substitution with NMR [37].

Amino acid sequence statistics in OI cases revealed that Ala and Ser mutations had the mildest phenotypes, whereas the homotrimer substitution yielded similar results [15]. The order of different Gly replacements in peptides can be represented as Ala < Ser < Arg < Val < Asp. This study confirms that substitutions introduced in individual chains has led to the same trend. Since the formation of fiber bundles requires helical stacking [13,46], the large side-chain in the Gly → Arg substitution may not only destabilize triple helices but also disrupt the assembly of fibers. Due to the length and sequence complexity of whole collagen, the molecular mechanism of OI mutations should be far more complicated than the local distortions described with the host–guest study of the collagen model peptide [17]. However, our study provides a method for dynamic analysis of local distortion caused by OI mutations, which may lay the foundation for future long-chain collagen research.

## Figures and Tables

**Table 1:** Sequence of heterotrimer *abc.*

Chain	Sequence
*a*	YGPKGPKGPKGKOGPDGDOGDOGDOGPKGPKG
*b*	YGPDGDOGDODOGPDGKOGPDGPDGPDGDOG
*c*	YGKOGPDGPDGPKGKOGPKGKOGKOGKOGKOG

**Table 2:** Characterizations of different OI model systems.

Chain	Mutation	*T*_m_	Lost H-Bonds ^a^	*E_HB_* ^b^(kcal∙mol^−1^)	Compensation H-Bonds
*a*	*a17S*	15.78 ± 0.33	2	−1.65 ± 0.28	1
	*a17V*	/	2	−1.45 ± 0.66	/
	*a17D*	/	2	−1.73 ± 0.30	/
	*a17R*	/	3	−1.45 ± 0.61	/
*b*	*b17S*	16.67 ± 0.24	1	−1.70 ± 0.60	1
	*b17V*	/	3	−1.71 ± 0.62	/
	*b17D*	/	1	−1.68 ± 0.65	/
	*b17R*	/	1	−1.67 ± 0.70	/
*c*	*c17S*	15.89 ± 0.36	1	−1.76 ± 0.49	1
	*c17V*	/	1	−1.68 ± 0.66	/
	*c17D*	/	1	−1.71 ± 0.65	/
	*c17R*	/	1	−1.73 ± 0.66	/

^a^: An increase in H-bond energy of more than 10% is defined as lost. ^b^: The energy value is the average of 13th–18th H-bonds.

## Data Availability

Not applicable.

## Supplementary Materials

Supporting Information including details of chain b and c mutants. The following supporting information can be downloaded at: https://www.mdpi.com/article/10.3390/biom12081104/s1. Figure S1: EHB of H-bond in abc and after Gly of chain b and c 17th site was mutated.; Figure S2: Probability of H-bond binding of abc and change after Gly of chain b and c 17th site was mutated; Figure S3: Salt-bridge changes in a17S; Figure S4: The side length after chain b and c mutation in Cα triangle; Figure S5: The distribution of nitrogen and oxygen atoms in a broken H-bond in b17S and c17S; Figure S6: The distribution of mutant amino acid side-chains in chain b and c mutants.
