# Supplementary material for: Disrupting Effects of Osteogenesis Imperfecta Mutations Could Be Predicted by Local Hydrogen Bonding Energy
Source: Biomolecules. 2022 Aug 11;12(8):1104. doi: 10.3390/biom12081104 (PMC9405839; doi:10.3390/biom12081104)
Supplement: Supplementary file 1 [file biomolecules-12-01104-s001.zip › biomolecules-1841113-supplementary.pdf]

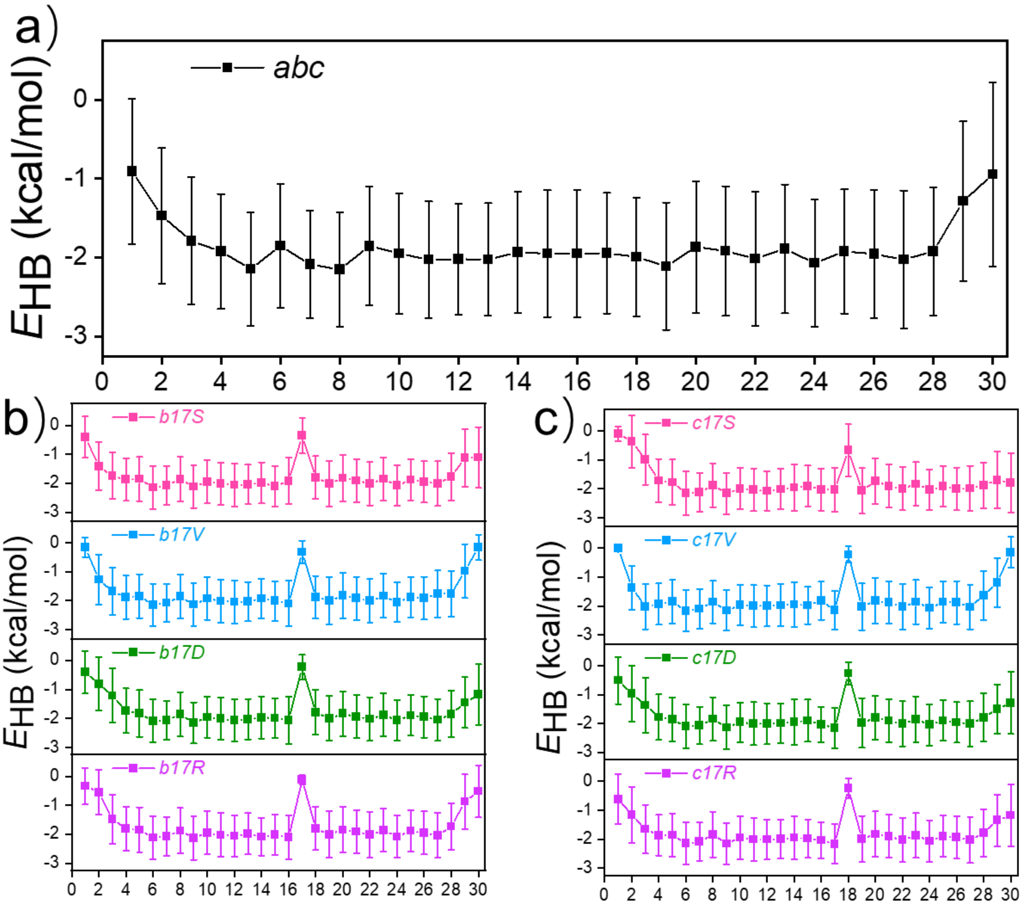

Figure S1 E_{HB} of H-bond in *abc* and after Gly of chain b and c 17th site was mutated. Index 1-30 is the position of each H-bond in the collagen.

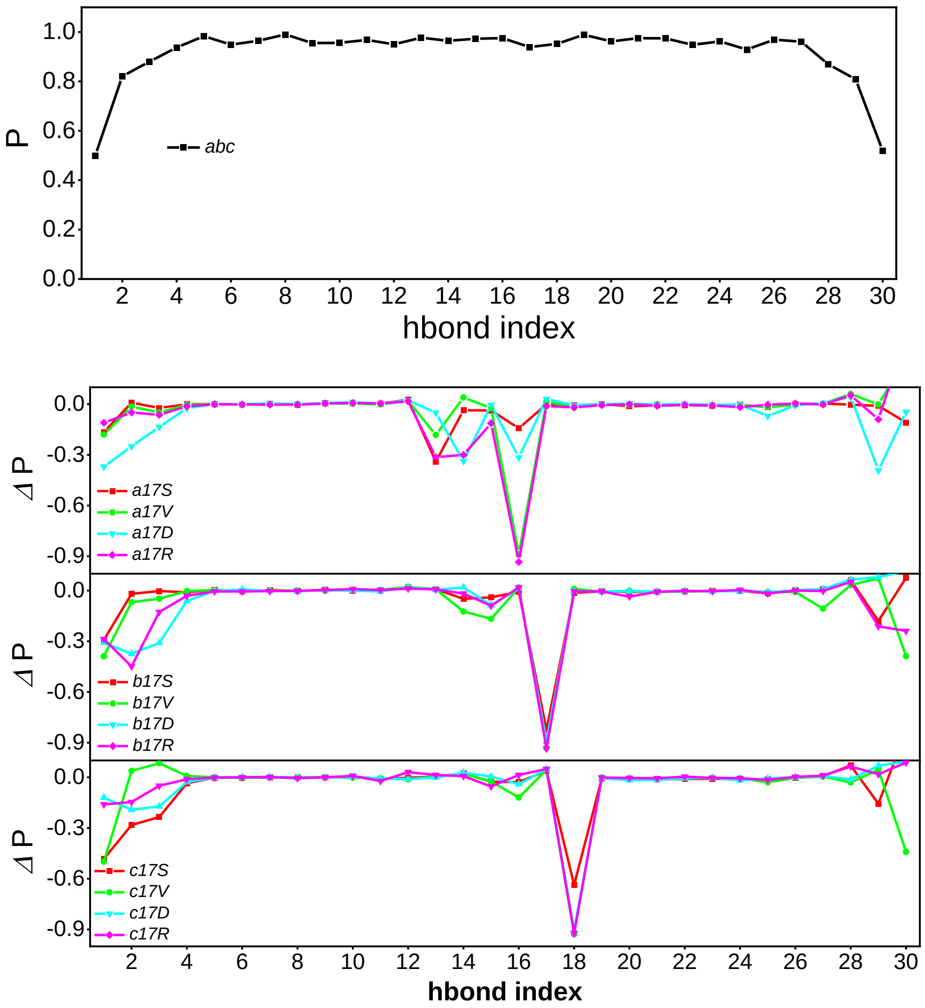

Figure S2 Probability of H-bond binding of *abc* and change after Gly of chain *b* and *c* 17th site was mutated. Index 1-30 is the position of each H-bond in the collagen.

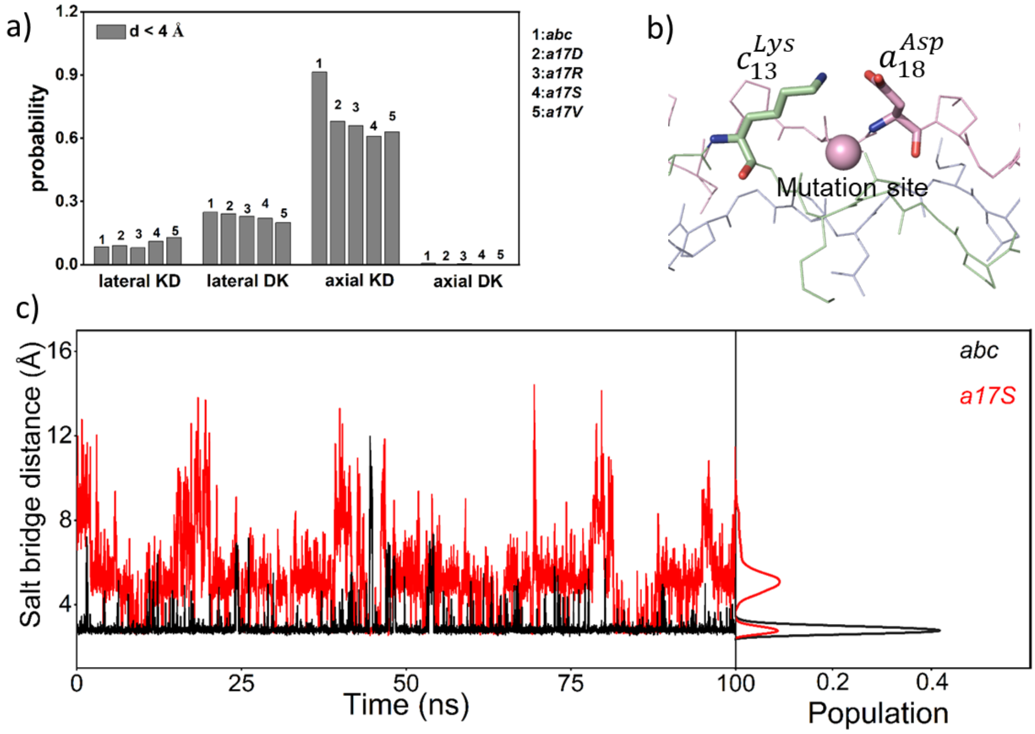

Figure S3 Salt-bridge changes. a) probability of salt-bridge distance change under 4 Å; b) mutation site around a broken salt-bridge formed by Lys and Asp; c) change in salt bridge distance over simulated time.

Since the substitution site is in the middle of the salt bridge, when the side chain is changed to a longer amino acid, NO distance of the salt bridge changed from one peak near 4 Å to two peaks in 4 Å and 6 Å, steric hindrance blocks the charge effect and the salt bridge is destroyed.

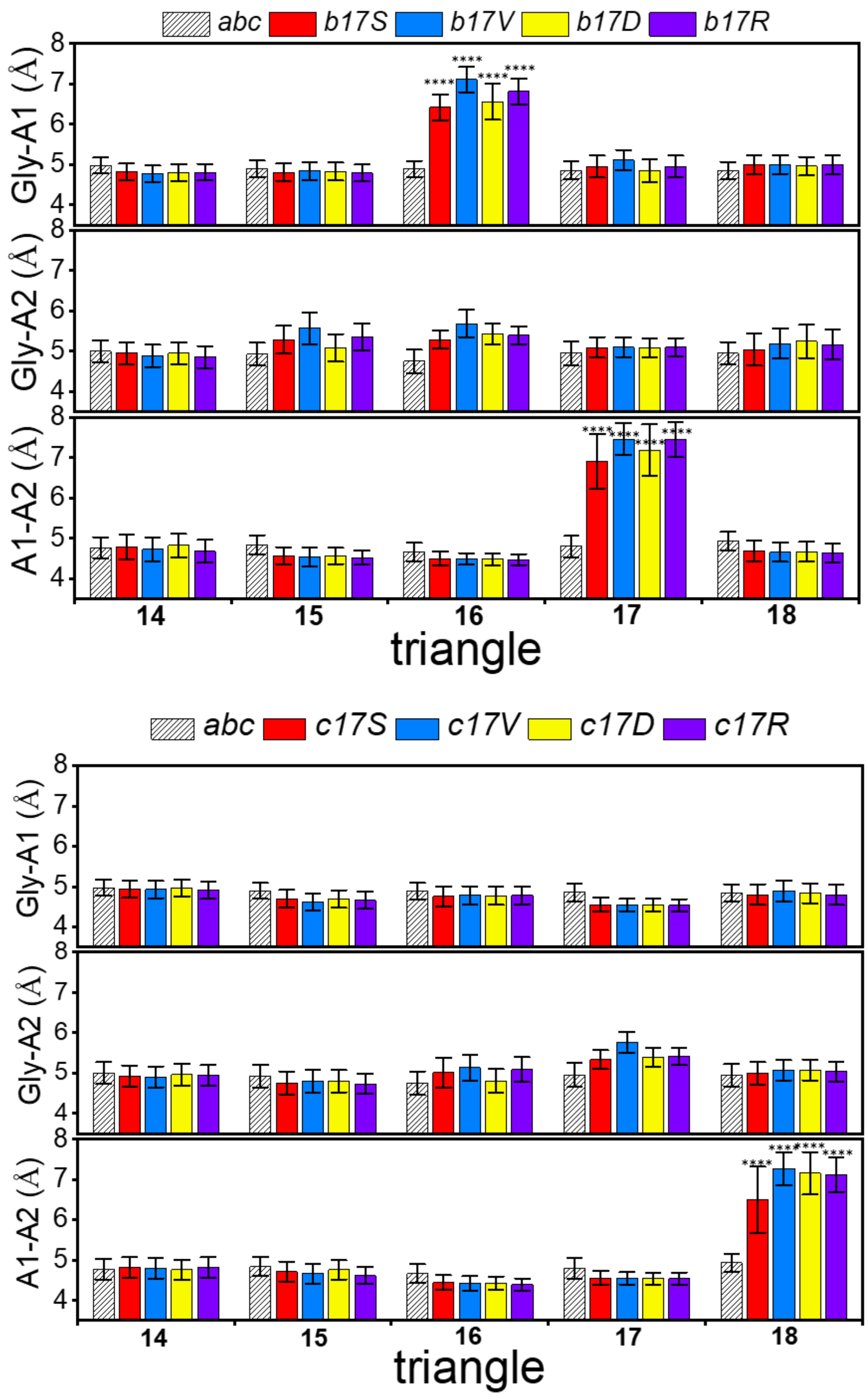

Figure S4 The side length after chain *b* and *c* mutation in Cα triangle.

Collagen have a stable state is triple helix, each distance of Cα-Cα in same plane is between 4.5 to 5 Å. All kind of mutant have similar tendency in broken these length of Cα. Indicate a strong decrease of collagen stability in consistent with loss of H-bond.

Statistical significance was calculated using one-wany ANOVA. Values are mean±SD.

**** P<0.0001, versus abc alone

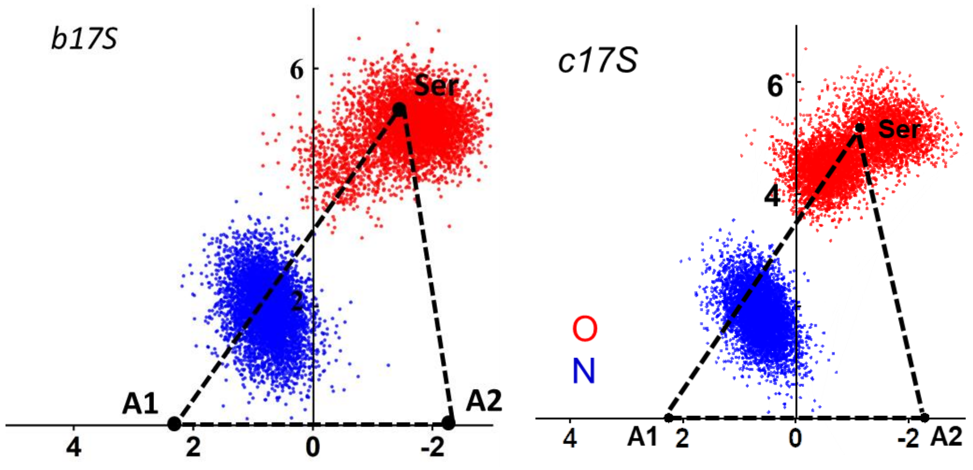

Figure S5 The distribution of nitrogen and oxygen atoms in a broken H-bond in b17S and c17S. Nitrogen atoms are shown in blue, and oxygen atoms are shown in red.

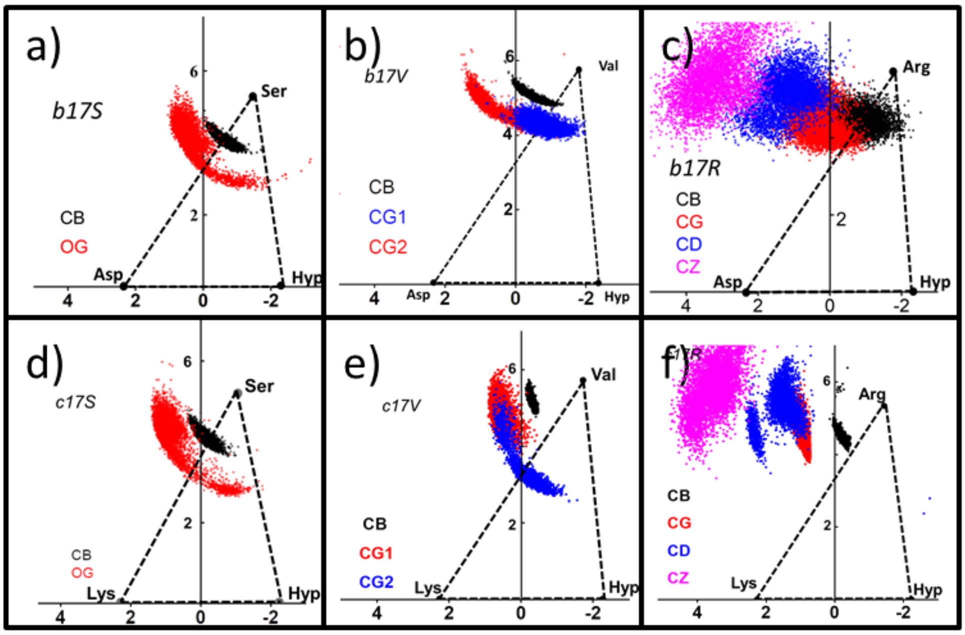

Figure S6 The distribution of mutant amino acid side-chains in chain b and c mutants. a-c) The side-chain distribution of b17S, b17V and b17R; d-f) The side-chain distribution of c17S, c17V and c17R
